# Developing muscarinic receptor M1 classification models utilizing transfer learning and generative AI techniques

**DOI:** 10.1038/s41598-025-00972-w

**Published:** 2025-05-12

**Authors:** Souvik Dey, Anders Wallqvist, Mohamed Diwan M. AbdulHameed

**Affiliations:** 1 Department of Defense Biotechnology High Performance Computing Software Applications Institute, Defense Health Agency Research and Development, Medical Research and Development Command, 504 Scott Street, Fort Detrick, MD 21702-5012 USA; 2 The Henry M. Jackson Foundation for the Advancement of Military Medicine, Inc., Bethesda, MD USA

**Keywords:** Computational biology and bioinformatics, Cheminformatics, Virtual screening

## Abstract

Muscarinic receptor subtype 1 (M1) is a G protein-coupled receptor (GPCR) and a key pharmacological target for peripheral neuropathy, chronic obstructive pulmonary disease, nerve agent exposures, and cognitive disorders. Screening and identifying compounds with potential to interact with M1 will aid in rational drug design for these disorders. In this work, we developed machine learning-based M1 classification models utilizing publicly available bioactivity data. As inactive compounds are rarely reported in the literature, we encountered the problem of imbalanced datasets. We investigated two strategies to overcome this bottleneck: *1*) transfer learning and *2*) using generative models to oversample the inactive class. Our analysis shows that these approaches reduced misclassification of the inactive class not only for M1 but also for other GPCR targets. Overall, we have developed classification models for M1 receptor that will enable rapid screening of large chemical databases and advance drug discovery.

**Supplementary Information:**

The online version contains supplementary material available at 10.1038/s41598-025-00972-w.

## Introduction

**Fig. 1:**
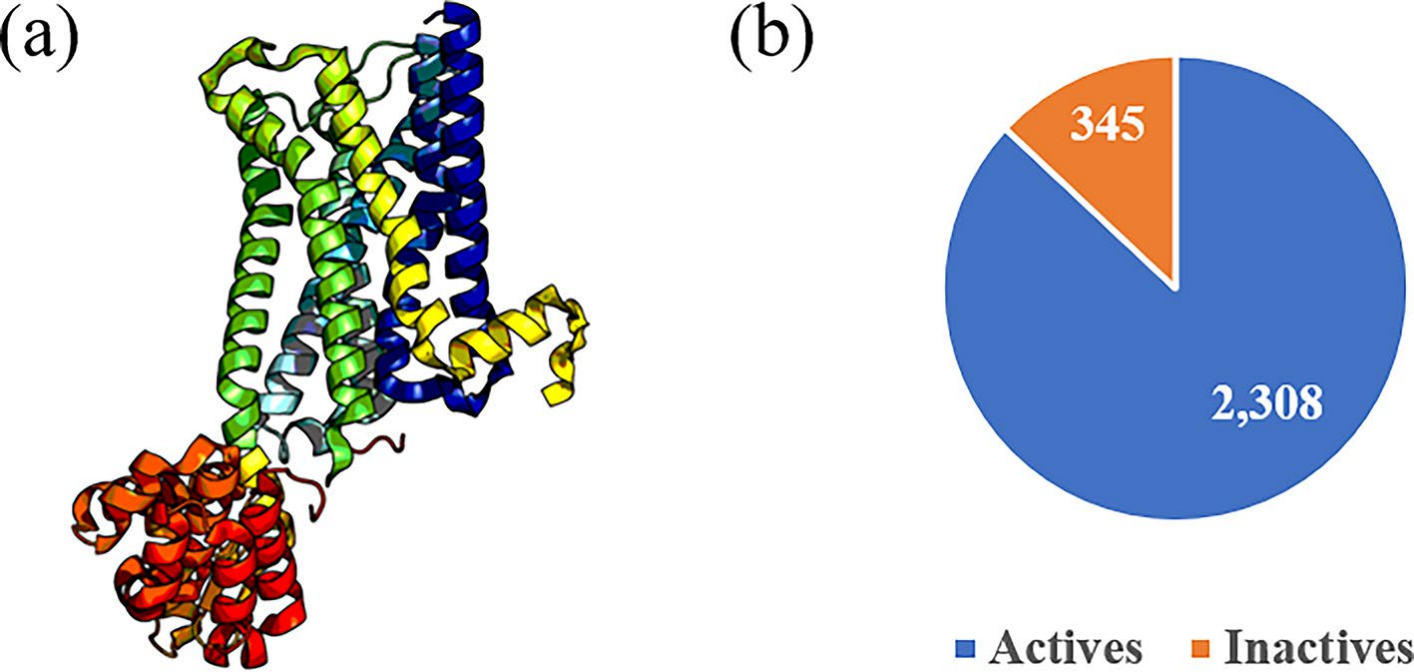
(**a**) Structure of muscarinic receptor subtype 1 (M1) (PDB ID: 5CXV). (**b**) Distribution of M1 bioactivity data collected from public sources, such as ChEMBL and BindingDB, considering a cutoff of 1 µM for actives and 10 µM for inactives.

Muscarinic receptors are a family of G protein-coupled receptors (GPCRs) that become activated in response to the neurotransmitter acetylcholine^1–3^. They play important roles in a variety of biological activities, including bladder muscle contraction, salivary gland secretion, and heart rate regulation, as well as control multiple cognitive processes^4–7^. However, overstimulation of muscarinic receptors, for instance by exposure to nerve agents, induces a cholinergic response that is characterized by narrowing of the airways, seizures, and even coma, making their inhibition relevant for military as well as civilian populations^8,9^. Recent studies have also identified muscarinic receptor subtype 1 (M1) (Fig. 1a) as a critical target for peripheral neuropathy, chronic obstructive pulmonary disease, and cognitive disorders. Pharmacological blockade of M1 receptors using antagonists has been proven to be effective in nerve regeneration, thereby reversing diabetes-, chemotherapy-, and HIV-induced neuropathies^10,11^.

The widespread distribution and impact of muscarinic receptors have prompted a multitude of efforts to discover novel therapeutics for M1. For example, atropine was found to be a nonspecific antagonist of muscarinic receptors and is now included in the current treatment for nerve-agent exposures^12–17^. Furthermore, using high-throughput screening (HTS), Merck identified a scaffold, benzyl quinolone carboxylic acid (BQCA), that can act as a positive allosteric modulator of M1^18^. Similarly, using HTS, Weaver et al. screened 63,656 compounds and identified 714 actives, three scaffolds of which were M1 selective. They further optimized these scaffolds and found that VU0255035 is an effective M1-selective antagonist^19^. Based on compounds that are similar to pirenzepine, using a scaffold-hopping approach, Millard et al. identified multiple muscarinic receptor antagonists with potential central nervous system activity^20^.

More recently, advances in artificial intelligence (AI) have enabled significant breakthroughs in drug discovery and development. AI-based models complement experimental studies by enabling screening of ultra-large libraries in the early stages of the drug discovery process. They also enable the identification of patterns in complex biological data that traditional Quantitative Structure Activity Relationship (QSAR) methods might overlook^21–25^. This increasing reliance on computational approaches is driven by the increased availability of bioactivities in public databases, such as ChEMBL, BindingDB, and PubChem, coupled with the expansion of the chemical space by generative models, such as recurrent neural networks, autoencoders, and transformers^26–35^. The improvements in computing power and advanced models have aided the drug discovery process, with several AI-predicted candidates advancing to clinical trials^36,37^.

Muscarinic receptors have been the focus of several computational studies as well. Tanczos et al. docked atropine and other similar compounds to wild-type and mutated rat M1, and the relative activities of these compounds agreed with previous experimental studies^38^. Montejo-López et al. docked 30 antagonists, 18 agonists, and 11 partial agonists and developed a QSAR model connecting the molecular volume of these compounds to their biological activities^39^. Mikurova et al. used free-energy calculations to develop models for calculating K_i_ values for 42 compounds against M1-M4^40^. However, all these previous computational approaches for M1 have either been structure-based or focused solely on one particular series of compounds for lead optimization, and there are no global machine learning models to effectively screen new compounds.

The first step in developing a machine learning model for M1 is to assemble the publicly available data from various bioactivity databases, such as ChEMBL and BindingDB. These aggregated public datasets are often imbalanced as inactives are rarely reported for dose-response studies^41^. Conversely, HTS studies, which test 100,000s of compounds against a target at a single dose and may only have ~ 100-1,000 active compounds, also create an imbalanced dataset, but in the opposite direction^42–44^. Models developed exclusively on these imbalanced datasets can be biased, with every query compound predicted to belong to the majority class, and such biased models are not practically useful for screening large chemical libraries^45^. Therefore, it is imperative to implement corrective measures to address these imbalances and enhance the reliability of ligand-based virtual screening.

Several previous cheminformatics studies have attempted to address this issue, both from a data and an algorithm perspective^46^. The data-driven methods focused on either oversampling the minority class by generating synthetic data or undersampling the majority class using methods such as clustering or cleaning up noisy data that hinder classification^47–52^. For example, Idakwo et al. applied several undersampling and oversampling methods on the well-studied Tox21 datasets and showed that oversampling the minority class using synthetic minority oversampling technique (SMOTE) followed by data cleaning using edited nearest neighbor (ENN) outperformed the other methods, although the performance deteriorated once the imbalance became more prominent^52^. Conversely, the algorithm-based methods, which focus on modifying an existing method by increasing the penalty for misclassifying the minority class, have been less frequent in the realm of virtual screening. For example, Li et al. applied the granular support vector machine repetitive undersampling method (GSVM-RU) to extract the most informative majority class samples to build a support vector machine model on a highly imbalanced luciferase HTS dataset^53^.

In this work, we compiled public bioactivity data for M1 to construct deep-learning-based classification models. The initial dataset had a disparate number of actives compared to inactives (Fig. 1b). The models performed well during 10-fold cross validation, but performance worsened significantly for both scaffold-split and HTS test sets, highlighting the commonly encountered problem of imbalanced datasets affecting model performance. In order to address this data imbalance problem when building effective M1 classification models, we employed two different methods: *1*) developing a transfer-learning framework using additional GPCR bioactivities and *2*) using two generative models, i.e., recurrent neural network (RNN) and transformer-based REINVENT4, to generate additional inactive compounds. These methods contributed to improving both the active and inactive classification results using diverse test sets, including one imbalanced in the opposite direction. We also show the applicability of our methods to other GPCR datasets. Overall, our work provides a well-validated computational tool for screening new compounds against M1 and highlights important strategies to tackle the issue of class imbalance while developing target-specific classification models. We envision that adopting such techniques will help advance future drug discovery efforts.

## Methods

### Data curation

We compiled bioactivity data (EC_50_, IC_50_, and K_i_) for our target M1 from public databases, i.e., ChEMBL and BindingDB. We retrieved the compounds as Simplified Molecular Input Line Entry System (SMILES) strings, which we subsequently processed for validity, desalted, and standardized using the ChEMBL structure pipeline^54^. We excluded entries lacking absolute bioactivity values, except for compounds with values greater than the inactive cutoff, which we labelled as inactives. Our overall goal in this study is to predict the potential of a compound to interact with the M1 receptor, not to predict the implications of this binding interaction (for example, agonism or antagonism). Hence, we collected and combined all available M1 interaction data throughout this work.

We applied an activity cutoff of ≤ 1 µM for actives and ≥ 10 µM for inactives. We chose the cutoff to minimize the overlap between the two classes, based on the Tanimoto similarity (see Results and discussion section for a detailed explanation). After assigning classification labels, we removed duplicate SMILES within the same class. If a compound was found in both classes, both SMILES were excluded. Next, using RDKit^55^, we generated Morgan fingerprints^56^ from the SMILES strings, with a length of 1,024 bits and a bond radius of 2. We divided the dataset into training and test sets using an 80:20 split, employing a scaffold-split strategy^57^ in DeepChem^58^. This method clusters compounds with similar scaffolds into the same class, thus creating a more challenging test set for the model. In addition, to ensure that all duplicates were removed, we also checked for duplicate fingerprints among the training and test sets, and removed duplicate entries from the training set. The final cleaned dataset contained 1,844 actives and 275 inactives in the training set and 464 actives and 70 inactives in the scaffold-split test set (Fig. 1b). Additionally, we identified a public HTS assay for M1 in PubChem (AID: 588852) and applied the pre-processing steps of desalting, standardization, and duplicate removal to generate a HTS test set consisting of 4,516 actives and 345,301 inactives. We also collected a final test set of known M1 antagonists from DrugBank^59^. This set has only active compounds (Table 1). The details of the composition of our training and test sets are shown in Table 1. To check the reproducibility of our methods across other targets with imbalanced data, we repeated the same procedure on five randomly chosen imbalanced GPCR datasets. Four of the targets belonged to the same subfamily of class A/rhodopsin-like receptors as M1, beta-2 adrenergic receptor, adenosine receptor A2a, C-C chemokine receptor type 5, and gastrin/cholecystokinin type B receptor, while metabotropic glutamate receptor 5 belonged to class C. Adenosine receptor A2a even belonged to the same subgroup A18 as M1.

**Table 1:** Composition of the different datasets used in this study along with their source of origin.

Dataset (acronym)	Origin	No. of actives	No. of inactives
Original training set (baseline DNN)	ChEMBL^26^, BindingDB^27^	1,844	275
Training set with RNN-generated inactives (RNN)	ChEMBL, BindingDB, RNN^35^	1,844	1,879
Training set with REINVENT4-generated inactives (R4)	ChEMBL, BindingDB, REINVENT4^60^	1,844	1,816
Scaffold-split test set	ChEMBL, BindingDB	464	70
HTS test set	PubChem^31^	4,516	345,301
DrugBank test set	DrugBank^59^	33	0

### Model building

We developed models using four different approaches: Bernoulli Naïve Bayes^61^, random forest^62^, XGBoost^63^, and deep neural network (DNN)^64^. We implemented the first three models using scikit-learn^65^. We constructed the DNN in Python 3.11, utilizing the Keras package with a TensorFlow 2.15 backend^66^. We adapted the DNN architecture from a previous study^67^ and carried out hyperparameter optimization, the details of which are shown in Supplementary Table S1. We performed a 10-fold cross validation for each of the combinations and chose the best-performing architecture. The input layer contained 1,024 neurons, with each feature representing an entry from the 1,024-bit Morgan fingerprints, and the output layer consisted of a single neuron. Between these layers, the network included two fully connected hidden layers with 1,000 and 500 neurons. We employed the Adam optimizer^68^ and used binary cross-entropy as the loss function, with a learning rate of 0.001, batch size of 64, and 2,000 epochs. We also applied an early stopping criterion with a patience of 50 such that the calculations stopped when the loss function did not improve after 50 epochs. To mitigate overfitting, we implemented dropout regularization with a rate of 0.25 in each layer. We used the rectified linear unit (ReLU) activation function for all the layers except the output layer, where we applied a sigmoid activation function, as this was a classification task requiring output probabilities between 0 and 1^69^.

### Methods to address class imbalance

We used two different methods to address the issue of class imbalance in the M1 dataset (transfer learning from GPCR data and using two generative models, RNN and REINVENT4, to generate additional inactive compounds), and we explain each method below. Since we wanted to assess the impact of our dataset-balancing approaches, further tuning of the DNN hyperparameters was not performed again. We repeated the same procedures for the five additional targets as well.

#### Transfer learning from GPCR data

**Fig. 2:**
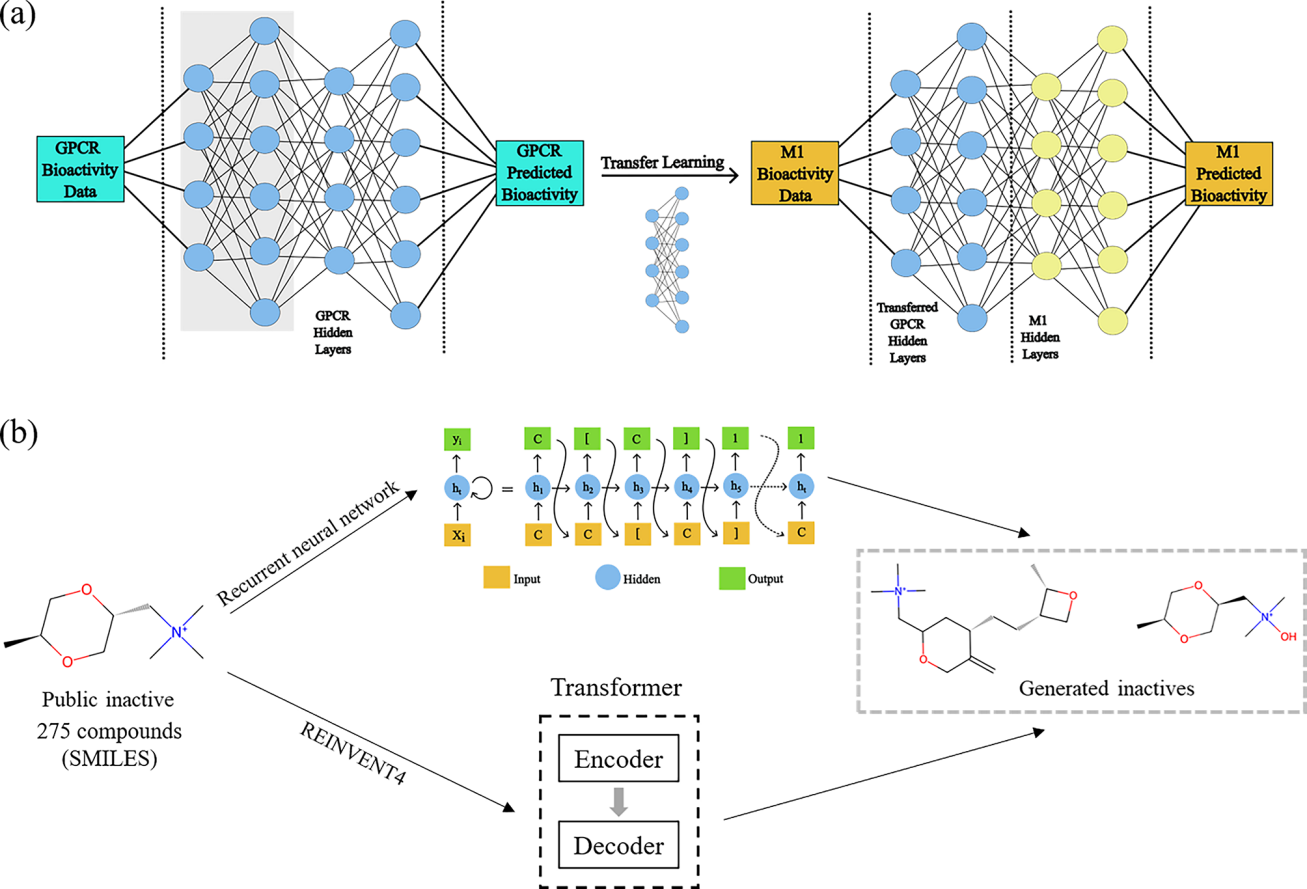
Overview of the two dataset-balancing methods: transfer learning and oversampling using generative models. (**a**) Transfer learning model architecture using G protein-coupled receptor (GPCR) bioactivity data from the GPCR-Ligand Association (GLASS) database^70^. (**b**) Overview of the two generative models. (Top) Recurrent neural network model architecture as adapted from Santana and Silva^35^. (Bottom) REINVENT4 model architecture as adapted from Loeffler et al.^60^.

We curated all the GPCR bioactivity data from the GPCR-Ligand Association (GLASS) database^70^, keeping the same criteria for classifying compounds, i.e., labeling compounds with EC_50_, IC_50_, or K_i_ values ≤ 1 µM as actives and ≥ 10 µM as inactives. In order to prevent data leakage, we removed compounds that were present in the M1 bioactivity dataset, resulting in a dataset comprised of 118,865 actives and 16,203 inactives. Similar to our M1 dataset, the GLASS database is also imbalanced, but we expect model improvement due to the exploration of additional chemical space coverage provided by the inactives. We first developed a DNN for the GPCR data following the same protocol outlined above. We then transferred the parameters from the first hidden layers into our initial DNN to create the transfer learning framework (Fig. 2a), without modifying any other component of the model^67^.

#### Using RNNs to oversample inactives

RNNs are among the various techniques used for generating new compounds, and they have been demonstrated to produce novel molecules with properties similar to those in a training set^28,34,35^. We employed a character-level RNN (CharRNN) specifically for this purpose, implementing it to oversample inactive compounds (Fig. 2b, top)^35^. This model learned the statistical structure of the SMILES syntax from a large number of SMILES and treated it as a language problem. We optimized the model parameters using maximum likelihood estimation and implemented the CharRNN using three-layered, long short-term memory (LSTM) RNN cells, each with a hidden dimension of 600. To mitigate overfitting, we inserted a dropout layer with a dropout probability of 0.2 between the intermediate layers. The output layer utilized a softmax activation function. We trained the model with a batch size of 64, using the Adam optimizer with a learning rate of 0.001 over 50 epochs. We evaluated the properties of the generated molecules according to the Molecular Sets (MOSES) benchmark^32^.

From this framework, we generated 200,000 compounds using a training set of 275 inactives and standardized them. We excluded SMILES strings that were shorter than the smallest compound in our training set, resulting in a final collection of 5,031 valid compounds. We clustered these RNN-generated compounds into 1,604 clusters and incorporated the cluster centroids into the training set, bringing the inactive set to 1,879 compounds.

#### Using REINVENT4 to oversample inactives

The RNN method was able to generate diverse compounds, but one of the major issues we faced was their low validity, which could possibly be attributed to our small training set. Consequently, we applied a more recent method, REINVENT4^60^, to generate M1 inactive compounds (Fig. 2b, bottom). Specifically, we utilized the mol2mol generator function^71^ in REINVENT4, which uses a transformer-based architecture. The model was pre-trained on 2.8 million pairs of molecules in the ChEMBL 28 database, originating from the same publication and containing the same Murcko scaffold, such that it could transform one of these molecules into another. The pre-trained model was available and fine-tuned on the M1 public inactives. Using a scaffold-based constraint allowed us to explore regions of the chemical space similar to the inactives, while also ensuring that the generated compounds were not too similar to the fine-tuning dataset. Our goal was to maximize exploration of new regions within the inactive space. Using a scaffold-based constraint also prevented us from adding molecules similar to our test set to avoid overfitting our model. We compared the compounds generated by the different REINVENT4 mol2mol foundation models,^71^ such as scaffold, medium/high similarity, and matched molecular pairs, and found that the compounds generated by scaffold constraints had the least average maximum Tanimoto similarity to the inactives. The Tanimoto similarity was calculated in RDKit using 1,024-bit Morgan fingerprints with a radius of 2. A detailed description of the transformer network can be found in an earlier study^72^.

We followed the same strategy as previously described in the section Using RNNs to oversample inactives to add the REINVENT4-generated compounds to our inactive dataset. Using the same training set of 275 inactives, we generated 8,919 compounds with this method. Following removal of compounds with SMILES strings shorter than the smallest compound in our training set, we combined the remaining compounds into 1,541 cluster centroids and included them in the inactive dataset, bringing their number to 1,816 compounds.

#### Comparison against traditional dataset-balancing approaches

We wanted to assess how our methods compare with a few traditional dataset-balancing approaches and implemented three methods paired to XGBoost: *1*) ENN^73^ for undersampling actives, *2*) SMOTE followed by ENN (SMOTE-ENN)^74^ to oversample inactives and clean the data, and *3*) KSMOTE^75^ to oversample inactives. We adapted these models from the Imbalanced-learn Python library from scikit-learn.

### Model evaluation

We performed model evaluation using 10-fold cross validation of the training set by randomly splitting the training set into 10 groups and leaving one of them out in each iteration. Due to the imbalanced nature of the data, we used a stratified split and kept the ratio between the active and inactive compounds constant across each fold (Supplementary Figure S1). For each of the 10 iterations, we calculated sensitivity, specificity, area under the receiver operating characteristic curve (ROC AUC), Matthews correlation coefficient (MCC), and G-mean (geometric mean between sensitivity and specificity). The test sets consisted of scaffold-split, HTS, and DrugBank datasets. We evaluated these datasets using the same metrics during each of the 10 cross-validation iterations. These parameters are defined as follows:

1 $${\text{Sensitivity}}=\frac{{\text{TP}}}{{\text{TP}}+{\text{FN}}},$$

2 $$\text{Specificity}=\frac{{\text{TN}}}{{\text{TN}}+{\text{FP}}},$$

3 $${\text{MCC}}=\frac{{\text{TP}}\cdot{\text{TN}} - {\text{FP}}\cdot{\text{FN}}}{\sqrt{{\text{(TP}} + {\text{FP}})\cdot({\text{TP}}+{\text{FN}})\cdot({\text{TN}}+{\text{FP}})\cdot({\text{TN}}+{\text{FN)}}}}$$

where TP represents true positive, TN denotes true negative, FP represents false positive, and FN denotes false negative. Given the need to compare performance across multiple models, and the possibility that one model may outperform the others by chance, we conducted a detailed statistical analysis using Friedman’s test^76^. This non-parametric test, analogous to ANOVA, can detect differences across multiple datasets. To further identify which models contributed to significant performance differences, we employed the Conover-Friedman post-hoc test on three metrics, i.e., MCC, ROC AUC, and G-Mean^77^. We considered a model to be significantly better than another only when the Conover-Friedman test was satisfied for each of the three metrics.

## Results and discussion

In this study, we developed a classification model to predict the potential of compounds to interact with muscarinic receptor M1. Recognizing the inherent imbalance in the M1 dataset, we employed two strategies to address this issue: transferring model parameters from a DNN trained on GPCR data and oversampling inactives by incorporating two generative models, i.e., RNN and REINVENT4. We validated our findings using a 10-fold cross validation of the training set and scaffold-split, HTS, and DrugBank test sets.

### Evaluation of classification cutoffs and their impact on data curation

We carried out a detailed analysis of the M1 bioactivity distribution which revealed a bias towards lower activity values (Supplementary Figure S2). While it is a standard practice to use a fixed cutoff, such as 1 or 10 µM, to distinguish between activity classes, we opted for a more refined approach to minimize class overlap. We designated an active cutoff at 1 µM and varied the inactive cutoff from 2 to 10 µM to exclude samples with ambiguous classifications. We noticed that as we increased the inactive cutoff, there was a reduction in the overlap between the active and inactive compounds, measured by a maximum Tanimoto similarity greater than 0.5 (Table 2). This also led to a small increase in the number of active compounds, even though their cutoff remained fixed. We settled for an inactive cutoff of 10 µM. Choosing a lower cutoff would have led to assigning inactive labels to many compounds that resembled the active class, affecting the built model. Moreover, previous studies have also shown that using a binary classification cutoff leads to better model performance as compared to a single cutoff^78,79^.

**Table 2:** Variation of the number of compounds in the active dataset showing overlap (indicated by max Tanimoto similarity > 0.5) with increasing inactive cutoff.

Active cutoff (µM)	Inactive cutoff (µM)	No. of actives	No. of inactives	Max Tanimoto similarity > 0.5
1	1	2,219	1,591	1,112
1	2	2,256	1,311	847
1	3	2,279	1,181	737
1	4	2,285	1,074	671
1	5	2,288	1,007	611
1	6	2,296	955	604
1	7	2,299	915	581
1	8	2,302	874	574
1	9	2,306	852	548
1	10	2,308	345	359

Moreover, we also evaluated whether our approach of utilizing all available data and data integration from multiple assay types led to addition of noise and decreased model performance^80^. We evaluated our approach by creating datasets that included only IC_50_ or K_i_ values and re-ran the models. The IC_50_ dataset consisted of 586 actives and 95 inactives, while the K_i_ dataset contained 837 actives and 149 inactives. We did not observe any significant improvement in classification performance for M1 (Supplementary Figure S3 and Supplementary Table S2) during 10-fold cross validation.

### Analysis of the molecular properties of active and inactive compounds interacting with the muscarinic receptor M1

**Fig. 3:**
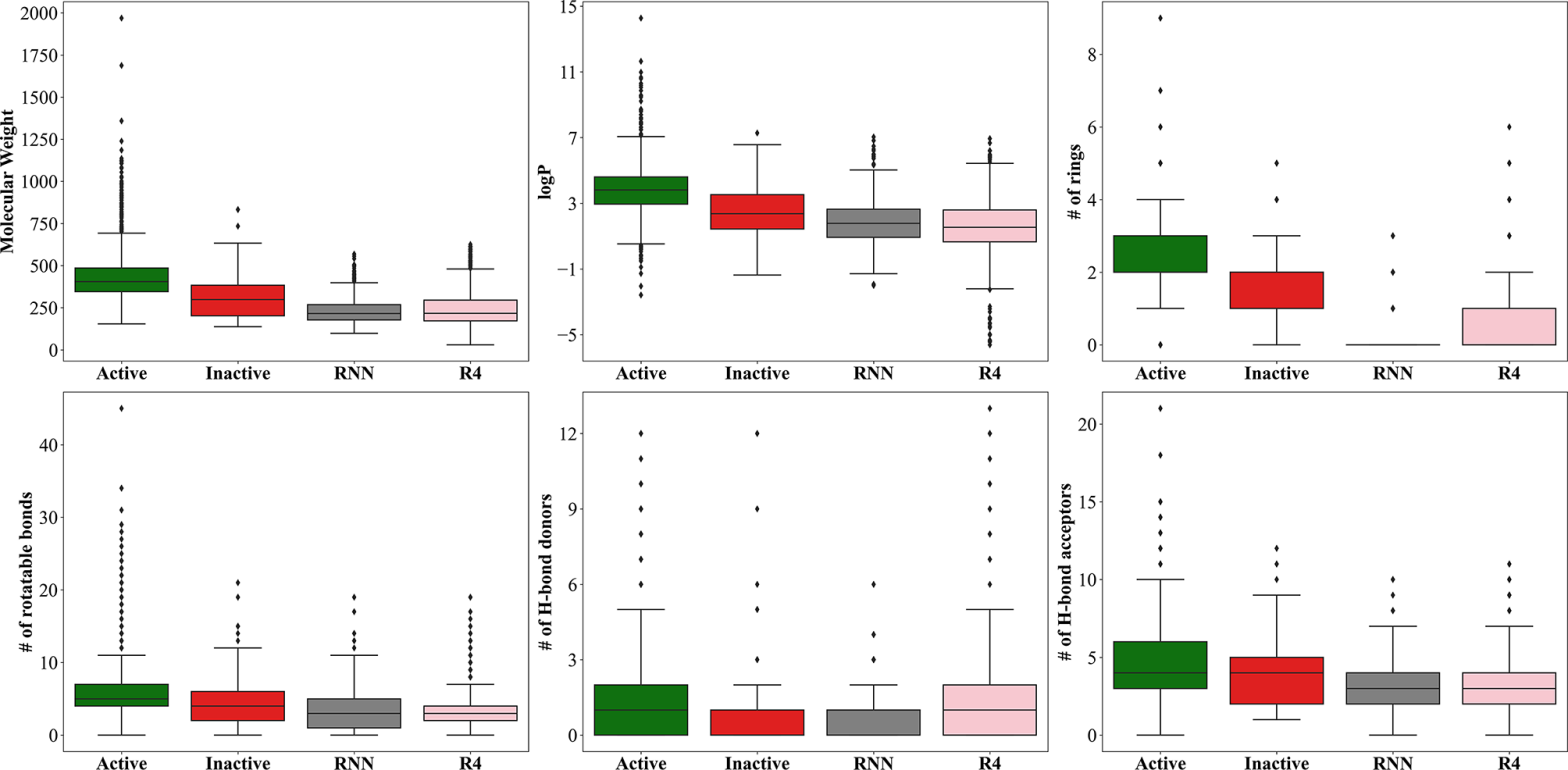
Distribution of six important physicochemical properties and their comparison among the active, inactive, recurrent neural network (RNN)-generated inactive, and REINVENT4 (R4)-generated inactive compounds. logP, logarithm of the octanol-water partition coefficient.

We calculated six well-characterized physicochemical descriptors—molecular weight, logarithm of the octanol-water partition coefficient, number of rings, rotatable bonds, and hydrogen bond donors and acceptors—for both active and inactive compounds using RDKit (Fig. 3). We treated inactive compounds generated by the RNN and REINVENT4 as separate classes. A comparison of the distribution of these descriptors revealed that the inactives generally had lower molecular weight, reduced lipophilicity, fewer rings, and slightly fewer rotatable bonds as compared to actives. However, due to the shear inequality of the class sizes, we were unsure whether these differences were significant.

The RNN-generated inactives exhibited a narrower distribution, with more similarity to the public inactives, albeit with many outliers, and were consistently lower. We noted that the RNNs had difficulty in generating ring-containing structures—only 22% of the generated compounds contained at least one ring—indicating potential challenges in capturing long-term dependencies. This limitation may originate from the small size of the training set, which included only 275 inactive compounds, compared to studies focused on larger datasets, such as the ZINC clean leads^32^ or GDB-13^81^. A closer look at the properties of these generated compounds (Supplementary Table S3) supported this observation, with a validity rate of only 3%. Efforts to generate additional compounds did not result in proportional increases in validity. Nevertheless, despite the low validity, the generated compounds were unique, novel, and diverse.

Due to limitations in validity and ring formation observed with compounds generated using the RNN model, we explored an alternative generative model REINVENT4, which uses a transformer-based architecture. It is trained on pairs of molecules containing same scaffolds and equipped to explore similar regions of chemical space as the public M1 inactives. Compounds generated by REINVENT4 exhibited superior validity, achieving 100%, as well as enhanced uniqueness. However, due to the controlled nature of their generation, they had a higher similarity to their nearest neighbor. Additionally, they displayed a slightly broader distribution of physicochemical properties and were able to overcome the ring generation problem faced by RNN-generated compounds. Compared to publicly available inactive compounds, the REINVENT4-generated compounds exhibited similar physicochemical profiles, with the exception of a reduction in the number of rings.

**Fig. 4:**
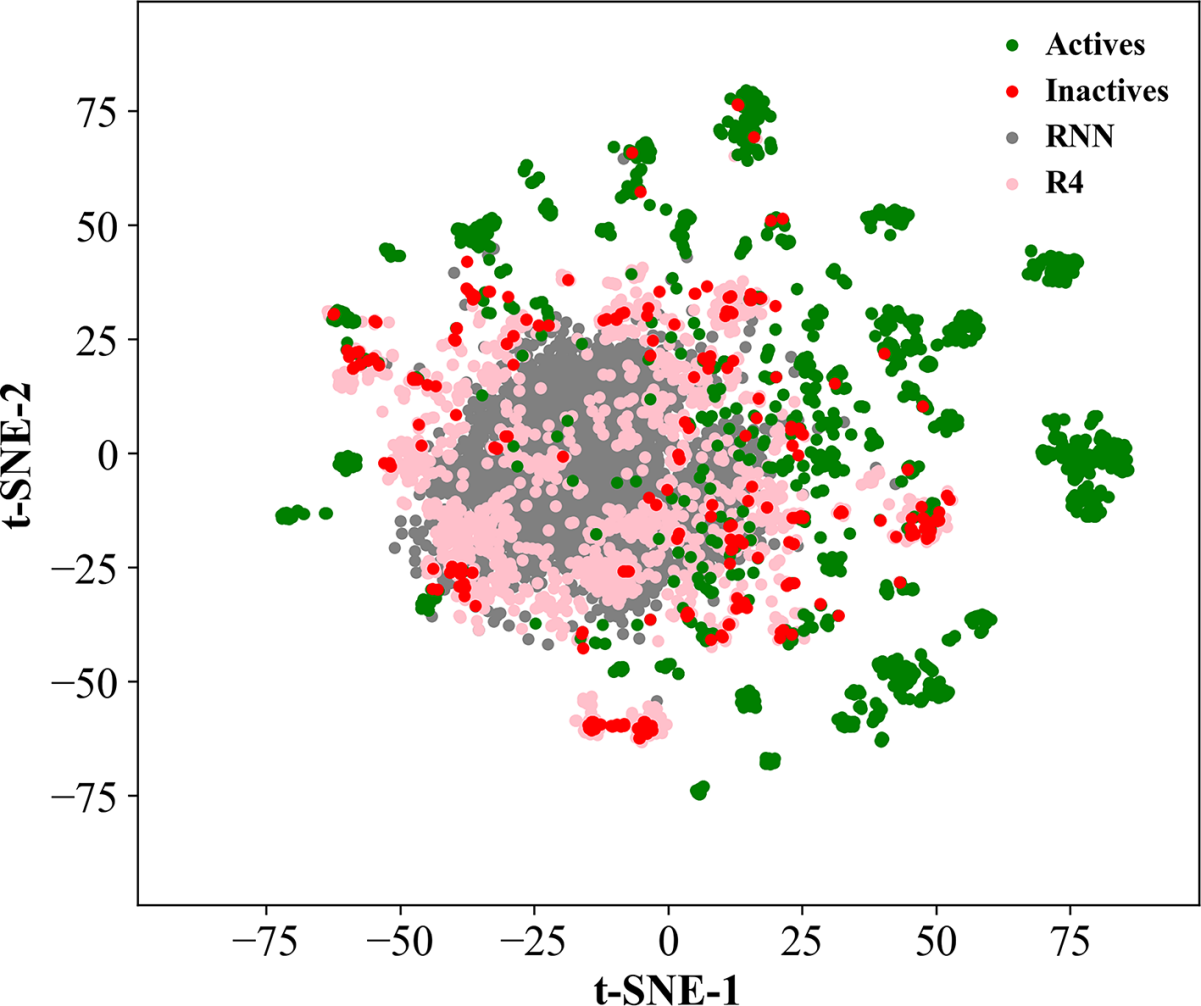
t-SNE plots of the chemical space occupied by the active (green dots), inactive (red dots), recurrent neural network (RNN)-generated inactive (grey dots), and REINVENT4 (R4)-generated inactive compounds (pink dots).

To further evaluate the diversity of the compounds, we constructed a t-distributed stochastic neighbor embedding (t-SNE) plot in scikit-learn using 1,024-bit Morgan fingerprints with a radius of 2 (Fig. 4). The chemical space displayed a broad distribution, with the RNN and REINVENT4 inactives occupying the central region and the public actives and inactives distributed more peripherally. We observed considerable overlap between the public actives and inactives, whereas compounds generated by REINVENT4 showed more overlap with the public inactives as compared to RNN, with overlaps being seen in almost all unique scaffolds. However, both methods missed sampling a few inactives that were closer to the public actives. The overlap between the RNN- and REINVENT4-generated compounds and the public actives was minimal.

### Model evaluation by cross validation

We conducted our initial model evaluation using 10-fold cross validation of the training set. We tested four different models—Naïve Bayes, random forest, XGBoost, and DNN—using the cross-validation analysis (Supplementary Table S4). All models showed strong performance in predicting both active and inactive compounds accurately. Sensitivity values were close to 1, which is expected for models trained on an imbalanced dataset. Specificity was also high, with Naïve Bayes achieving an average of 0.91 and DNN reaching 0.80. MCC, which is considered a robust indicator of model performance on imbalanced datasets^82^, also reached a very high value, with values as high as 0.83 for DNN. Additionally, the ROC AUC and G-Mean values were notably high across all models. A close comparison of the four models revealed that Naïve Bayes and random forest slightly underperformed (Supplementary Figure S4) on this cross-validation dataset. XGBoost and DNN both produced very similar metrics, but we selected DNN as our baseline for further evaluation, as we built a transfer learning protocol using this method.

**Fig. 5:**
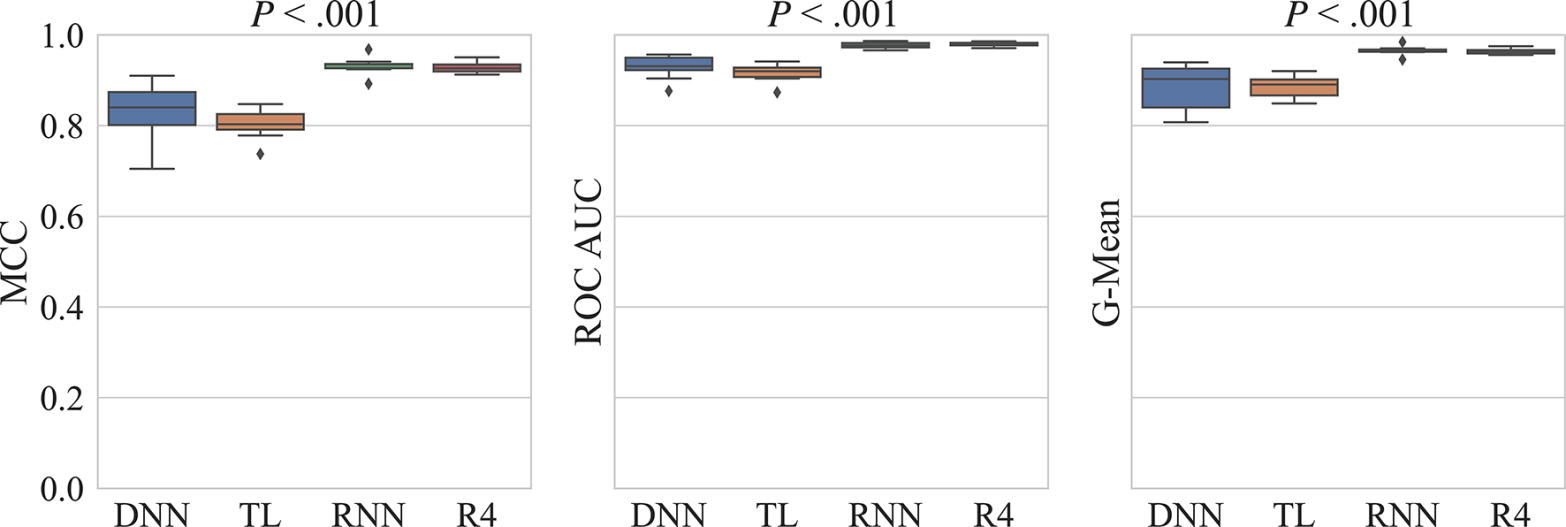
Boxplots showing comparisons of the baseline DNN against the two dataset-balancing methods (TL and RNN/R4) during 10-fold cross validation for MCC, ROC AUC, and G-Mean. The *P*-value determined using Friedman’s test is shown above each plot. MCC: Matthews correlation coefficient; ROC AUC: area under the receiver operating characteristic curve; G-Mean: geometric mean of sensitivity and specificity; DNN: deep neural network; TL: transfer learning; RNN: recurrent neural network; R4: REINVENT4.

The performance of the models trained on the imbalanced dataset was already reasonable, and balancing the dataset led to further improvements, especially after incorporating the additional inactives from the generative models (Fig. 5 and Supplementary Table S5). Sensitivity remained high even with the addition of more inactive compounds, with a notable increase in specificity, reaching an average of 0.96 when incorporating either RNN- or REINVENT4-generated inactives. The MCC, ROC AUC, and G-Mean of the RNN- and REINVENT4-addition models were significantly better than the other two models, as indicated by the low *P*-values determined using Friedman’s test (Fig. 5) and the Conover-Friedman test (Supplementary Figure S5).

### Model evaluation on test sets

**Fig. 6:**
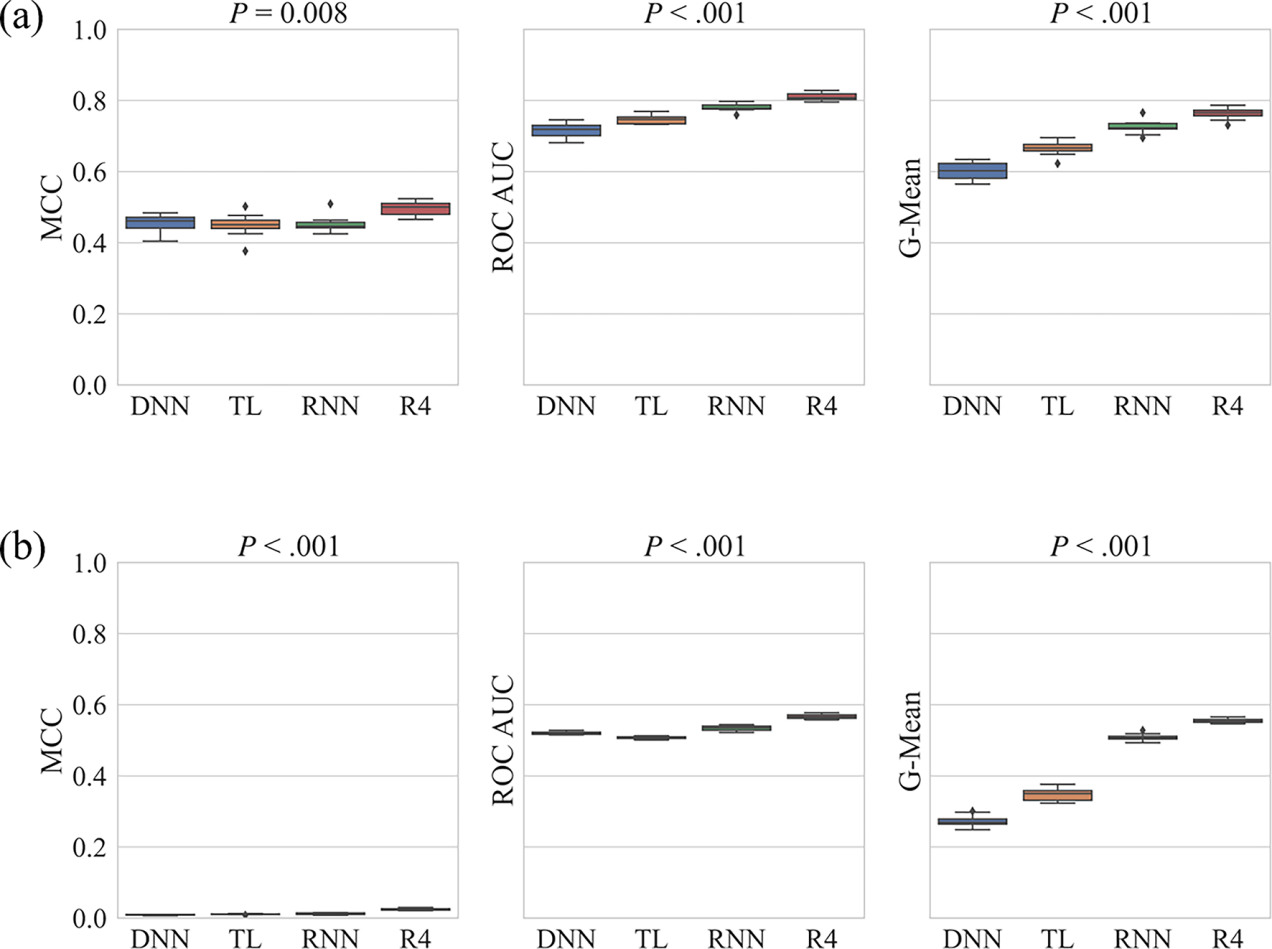
Boxplots showing comparisons of the baseline DNN against the two dataset-balancing methods (TL and RNN/R4) during (**a**) scaffold-split and (**b**) HTS test sets predictions for MCC, ROC AUC, and G-Mean. The *P*-value determined using Friedman’s test is shown above each panel. MCC: Matthews correlation coefficient; ROC AUC: area under the receiver operating characteristic curve; G-Mean: geometric mean of sensitivity and specificity; DNN: deep neural network; TL: transfer learning; RNN: recurrent neural network; R4: REINVENT4.

We achieved strong performance metrics for our models during cross validation, particularly when the training set was enhanced with RNN- or REINVENT4-generated inactives. However, our hyperparameters were tuned during this process, making the baseline DNN optimized for this dataset. Moreover, we used random splits to create the cross-validation folds. These factors are very likely to cause model overfitting, and a more challenging task for the models would be to predict M1 binding for compounds not encountered during training. We constructed the first test set using scaffold-split of the public data. While sensitivity remained close to 1 for our baseline DNN model, specificity dropped significantly from 0.81 during cross validation to 0.37 for this test set (Supplementary Table S6). This decline was accompanied by decreases in MCC, ROC AUC, and G-Mean, suggesting that the model was misclassifying a substantial number of inactive compounds. Efforts to balance the training set led to marked improvements in predicting this test set (Fig. 6a). Both ROC AUC and G-Mean improved from 0.72 to 0.75 and from 0.60 to 0.67, respectively, after we implemented transfer learning from the GLASS database, and they increased further to 0.81 and 0.76, respectively, following the incorporation of additional inactive compounds from REINVENT4. Of these two generative methods, REINVENT4 slightly outperformed RNN, probably because it had more valid and unique compounds (Supplementary Table S2). In fact, REINVENT4 significantly outperformed all other methods in all the three metrics (Supplementary Figure S6).

We then wanted to test the generalizability of our models in a new chemical space, so we opted to apply our models to predict the HTS test set. The baseline DNN model’s performance worsened even more significantly when predicting this dataset. HTS datasets typically are imbalanced in the opposite direction as compared to public datasets, and this was no exception, making them very challenging to begin with. Our baseline DNN model was not able to correctly identify the large number of inactive compounds, with specificity falling to a mere 0.08 (Supplementary Table S7). Sensitivity remained high, with average values reaching 0.94. Balancing the datasets improved specificity to 0.56 through REINVENT4-addition. Transfer learning and RNN-addition also improved specificity, and consequently G-Mean (Fig. 6b). However, these improvements were achieved at a cost of reduced sensitivity, decreasing to 0.56 upon REINVENT4-addition. MCC for this dataset was always very low, as even with a specificity of 0.56, the REINVENT4-addition approach misclassified 157,481 inactive compounds. Hence, when we compared two M1 test sets differing by orders of magnitude in size, we could not rely on MCC. A recent study also highlighted this same issue, showing that MCC is dependent on the ratio between the two classes and may be underestimated during extreme class imbalance^83^. ROC AUC was around 0.50 for most models, reaching its highest value of 0.57 upon REINVENT4-addition, indicating the near-random nature of the predictions. The trend of the two dataset-balancing methods was the same as seen during the earlier prediction of the scaffold-split test set. REINVENT4 had the best performance, followed closely by RNN, and transfer learning showed the least improvement compared to the baseline DNN model and slightly reduced ROC AUC (Supplementary Figure S7).

Finally, we applied the models to a manually collected set of M1 antagonists from DrugBank. The baseline DNN model performed exceptionally well on this single-class dataset, achieving an accuracy of 0.97 (Supplementary Table S8). This was expected considering the imbalanced nature of this model and an all-active test set, and we wanted to evaluate whether our dataset-balancing strategies led to a decrease in accuracy. However, even after we balanced the dataset, the three models continued to perform well, with the lowest accuracy being 0.92 for REINVENT4. Transfer learning was the best-performing method, correctly predicting all the M1 active compounds.

We further assessed the performance of our transfer learning and generative AI approaches for balancing datasets in comparison to three traditional techniques: ENN, SMOTE-ENN, and KSMOTE. We evaluated these methods on both the scaffold-split and HTS test sets. Among the three, SMOTE-ENN showed the best performance, achieving MCC and G-Mean values comparable to those obtained with REINVENT4-addition on the scaffold-split test set (Supplementary Table S6). However, all three methods performed poorly on the HTS test set, underscoring limitations in real-world applications (Supplementary Table S7). These differences likely arise from the inability of traditional techniques to effectively expand the chemical space, as they primarily generate new data points within existing regions and lack the capability to explore more diverse areas compared to generative models.

In a nutshell, our baseline model, built on an imbalanced dataset, showed excellent performance on the cross-validation set but struggled to replicate this success on the external test sets. The modifications we introduced to balance the training set not only improved the strong performance of the baseline model on the cross-validation dataset but also significantly improved its performance across the two external test sets, with the REINVENT4-addition model outperforming the other models in both cases.

### Model evaluation on other datasets

Finally, we evaluated our methods on other datasets to show that the improvement in model performance is not unique to M1 and that our approaches are applicable for any target. We used our methods on five imbalanced GPCR datasets for both 10-fold cross validation and scaffold-split test sets and observed significant improvement in model performance in four out of the five datasets (Supplementary Figures S8-S12). Among our methods, transfer learning was slightly inconsistent and led to a performance deterioration in a few cases, but both generative models, RNN and REINVENT4, offered a consistent enhancement. These results further demonstrate the generalizability of our methods.

## Conclusion

In this study, we developed machine learning models to screen new compounds against M1. Our initial model developed using public datasets was imbalanced, leading to a biased model, and we presented two major strategies to address this issue. The first strategy utilizes the widely adopted technique of transfer learning by building a model with GPCR data and transferring the model parameters. This method showed modest improvements for our M1 dataset, across both external test sets. Our second strategy leverages the use of generative models, a rapidly evolving field, to augment the inactive class. By generating a diverse set of inactive compounds using RNN and REINVENT4, we observed a notable improvement in all our test sets, with better performance than transfer learning. Of these two generative models, RNN struggled to capture long-term dependencies, potentially due to the small training set of 275 compounds, and had low validity. To counter this issue, we implemented REINVENT4, which worked well even on a small training set, generating slightly more diverse compounds covering most unique scaffolds, and we noticed an immediate improvement in performance. Collectively, the solutions we propose enhanced our M1 classification model across three diverse test sets, underscoring the importance of balancing training sets. These approaches are not limited to just M1 but could also be translated to other targets.

This study primarily focused on model development for the muscarinic M1 receptor. Future work will explore the utility of the models in evaluating potential interaction with repurposed drugs. The newly developed models could also be used for screening large chemical databases, such as the 65 billion synthesizable compounds from the Enamine REAL database in future drug discovery efforts. Current computational efforts in GPCR hit discovery are predominantly structure-based, and our models provide a complementary ligand-based approach to enhance virtual screening.

## Electronic supplementary material

Below is the link to the electronic supplementary material.

Supplementary Material 1

## Data Availability

The data employed to conduct our analysis are available on GitHub, at the following URL https://github.com/BHSAI/imbalanced_data_M1.
