# Supplementary material for: Developing muscarinic receptor M1 classification models utilizing transfer learning and generative AI techniques
Source: Sci Rep. 2025 May 12;15:16486. doi: 10.1038/s41598-025-00972-w (PMC12069682; doi:10.1038/s41598-025-00972-w)
Supplement: Supplementary file 1 — Supplementary Material 1 [file 41598_2025_972_MOESM1_ESM.pdf]

Supplementary Information

Developing muscarinic receptor M1 classification models utilizing transfer learning and generative AI techniques

Souvik Dey[1,2], Anders Wallqvist[1], and Mohamed Diwan M. AbdulHameed[1,2]**

[1]Department of Defense Biotechnology High Performance Computing Software Applications Institute, Defense Health Agency Research & Development, Medical Research and Development Command, Fort Detrick, MD, USA

[2]The Henry M. Jackson Foundation for the Advancement of Military Medicine, Inc., Bethesda, MD, USA

*Corresponding Authors:

AW E-mail: sven.a.wallqvist.civ@health.mil
MDMA E-mail: mabdulhameed@bhsai.org

DoD Biotechnology High Performance Computing Software Applications Institute
Defense Health Agency Research & Development
Medical Research and Development Command
ATTN: FCMR-TT, 504 Scott Street
Fort Detrick, MD 21702-5012
Tel: (301) 619-1989; Fax: (301) 619-1983

Table S1. Details of our hyperparameter optimization protocol

**Hyperparameters**	**Tuning space**	**Optimal choice**
Number of hidden layers	1/2/3	2
Number of neurons	100/500/1,000/2,000/4,000/6,000 (1 hidden layer)<br>1,000/2,000/4,000 and 100/500/1,000/2,000 (2 hidden layers)<br>1,000/2,000, 500/1,000, and 100/500 (3 hidden layers)	1,000 and 500
Dropout rates	0.05/0.10/0.25/0.50	0.25
Activation functions	ReLU/Sigmoid/tanh/Leaky ReLU	ReLU
Learning rate	0.01/0.001	0.001

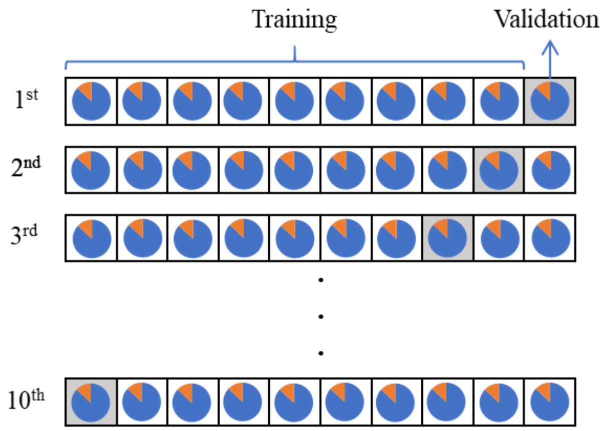

Figure S1. Concept of the stratified 10-fold cross validation.

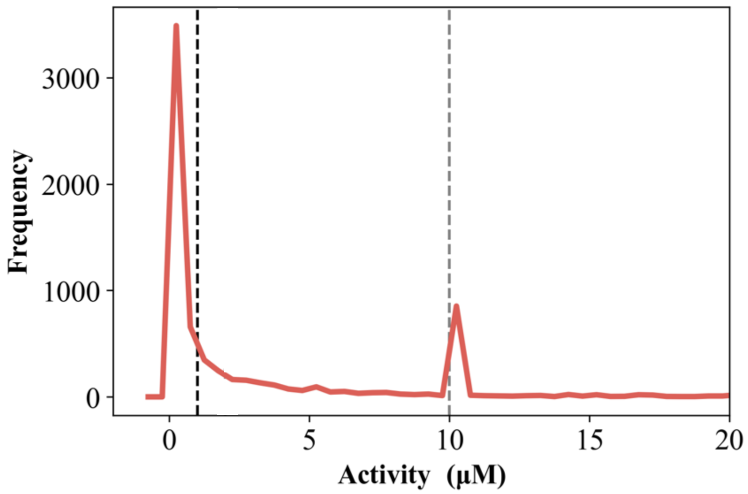

Figure S2. Histogram of the M1 bioactivity data from public sources. The inactive (10 μM) and active (1 μM) cutoffs are shown as gray and black dashed lines, respectively.

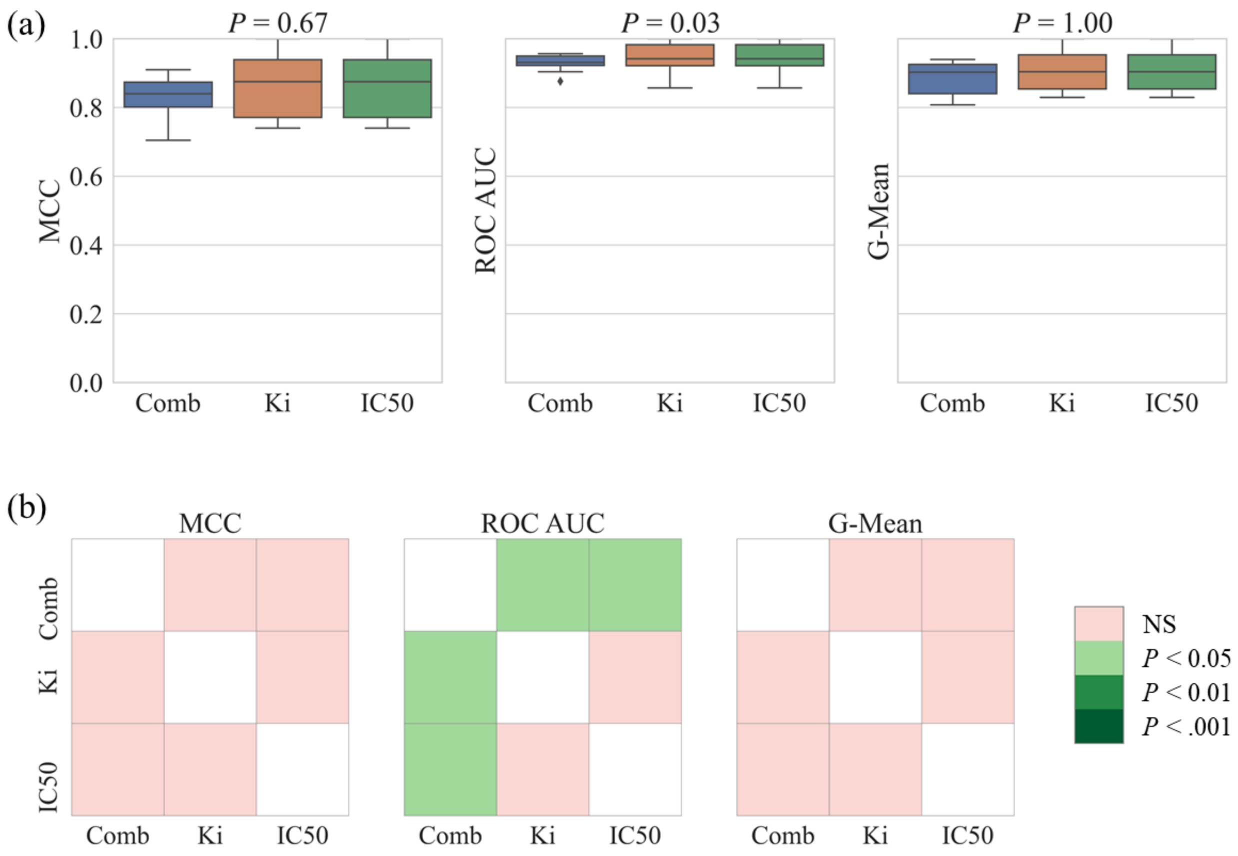

Figure S3. Statistical comparisons of the performance of different datasets containing only IC_{50} and K_i against the combined dataset during 10-fold cross validation. (a) Boxplots showing comparisons of different datasets (Combined, Only K_i, and Only IC_{50}) comparing MCC, ROC AUC, and G-Mean. The *P*-value determined using Friedman's test is shown above each panel. (b) Sign plots showing results from the Conover-Friedman test. MCC: Matthews correlation coefficient; ROC AUC: area under the receiver operating characteristic curve; G-Mean: geometric mean of sensitivity and specificity; NS: not significant.

Table S2. Performance of different datasets containing only IC_{50} and K_i against the combined dataset during 10-fold cross validation of the training set

Model	Sensitivity	Specificity	MCC	ROC AUC	G-Mean
DNN-Combined	0.99 (0.01)	0.80 (0.09)	0.83 (0.06)	0.93 (0.03)	0.89 (0.05)
DNN-Only K_i	0.99 (0.01)	0.81 (0.06)	0.87 (0.04)	0.92 (0.03)	0.90 (0.03)
DNN-Only IC_{50}	0.99 (0.02)	0.84 (0.11)	0.86 (0.09)	0.94 (0.05)	0.91 (0.06)

Data are presented as mean (standard deviation). MCC: Matthews correlation coefficient; ROC AUC: area under the receiver operating characteristic curve; G-Mean: geometric mean of sensitivity and specificity; DNN: deep neural network.

Table S3. Properties of the generated inactives as defined in MOSES[1]

Property	RNN	R4
Valid	0.03	1.00
Unique@1k	0.96	1.00
Unique@10k	0.89	1.00
Novelty	0.99	0.99
Similarity to nearest neighbor	0.27	0.47
Internal diversity	0.89	0.89

RNN: recurrent neural network; R4: REINVENT4.

Table S4. Performance of different models on the imbalanced dataset during 10-fold cross validation

Model	Sensitivity	Specificity	MCC	ROC AUC	G-Mean
Naïve Bayes	0.89 (0.02)	0.91 (0.05)	0.66 (0.05)	0.90 (0.03)	0.90 (0.03)
Random forest	0.99 (0.01)	0.73 (0.11)	0.79 (0.06)	0.86 (0.05)	0.85 (0.06)
XGBoost	0.98 (0.01)	0.79 (0.11)	0.81 (0.07)	0.89 (0.05)	0.88 (0.06)
DNN	0.99 (0.01)	0.80 (0.09)	0.83 (0.06)	0.93 (0.03)	0.89 (0.05)

Data are presented as mean (standard deviation). MCC: Matthews correlation coefficient; ROC AUC: area under the receiver operating characteristic curve; G-Mean: geometric mean of sensitivity and specificity; DNN: deep neural network.

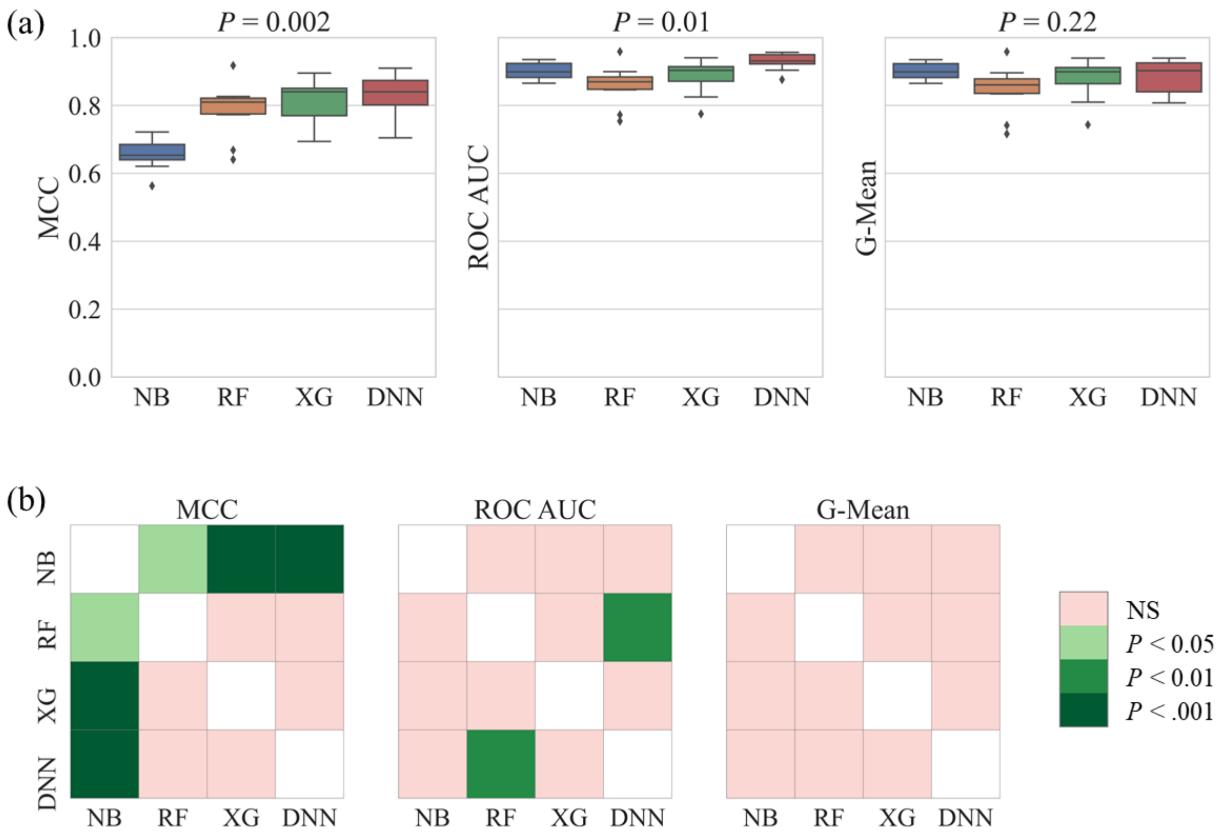

Figure S4. Statistical comparisons of the performance of different models on the imbalanced dataset during 10-fold cross validation. (a) Boxplots showing comparisons of different methods comparing MCC, ROC AUC, and G-Mean. The *P*-value determined using Friedman's test is shown above each panel. (b) Sign plots showing the results from the Conover-Friedman test. MCC: Matthews correlation coefficient; ROC AUC: area under the receiver operating characteristic curve; G-Mean: geometric mean of sensitivity and specificity; DNN: deep neural network; XG: XGBoost; RF: random forest; NB: Naïve Bayes; NS: not significant.

Table S5. Performance of the DNN compared with the two proposed dataset-balancing methods (TL and RNN/R4) during 10-fold cross validation

Model	Sensitivity	Specificity	MCC	ROC AUC	G-Mean
DNN	0.99 (0.01)	0.80 (0.09)	0.83 (0.06)	0.93 (0.03)	0.89 (0.05)
DNN-TL	0.98 (0.01)	0.80 (0.05)	0.80 (0.03)	0.92 (0.02)	0.89 (0.03)
DNN-RNN	0.97 (0.01)	0.96 (0.01)	0.93 (0.02)	0.98 (0.01)	0.97 (0.01)
DNN-R4	0.97 (0.01)	0.96 (0.01)	0.93 (0.01)	0.98 (0.01)	0.96 (0.01)

Data are presented as mean (standard deviation). MCC: Matthews correlation coefficient; ROC AUC: area under the receiver operating characteristic curve; G-Mean: geometric mean of sensitivity and specificity; DNN: deep neural network; TL: transfer learning; RNN: recurrent neural network; R4: REINVENT4.

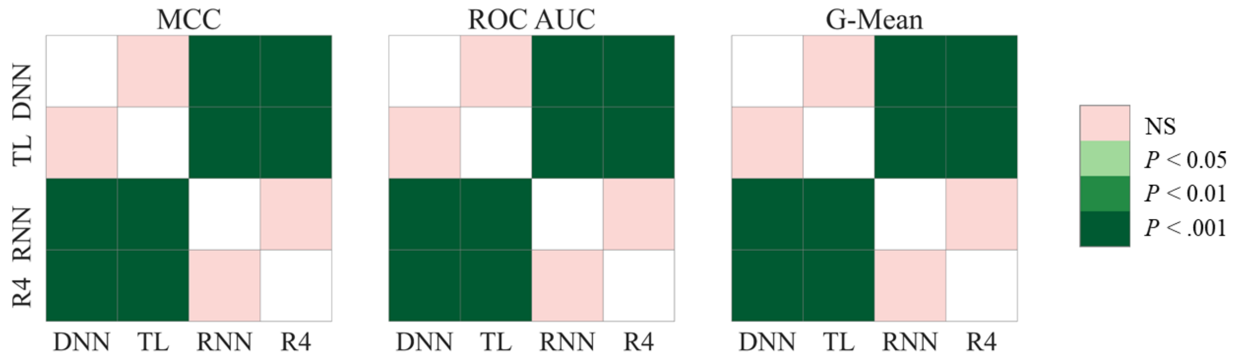

Figure S5. Statistical comparison of the DNN against the two dataset-balancing methods (TL and RNN/R4) during 10-fold cross validation using sign plots showing results from the Conover-Friedman test for MCC, ROC AUC, and G-Mean. MCC: Matthews correlation coefficient; ROC AUC: area under the receiver operating characteristic curve; G-Mean: geometric mean of sensitivity and specificity; DNN: deep neural network; TL: transfer learning; RNN: recurrent neural network; R4: REINVENT4; NS: not significant.

Table S6. Performance of the DNN compared with the two proposed dataset-balancing methods (TL and RNN/R4) and three traditional approaches during scaffold-split-generated test set prediction

Model	Sensitivity	Specificity	MCC	ROC AUC	G-Mean
DNN	0.97 (0.00)	0.37 (0.03)	0.45 (0.03)	0.72 (0.02)	0.60 (0.03)
DNN-TL	0.95 (0.01)	0.47 (0.04)	0.45 (0.02)	0.75 (0.01)	0.67 (0.02)
DNN-RNN	0.91 (0.01)	0.58 (0.03)	0.45 (0.02)	0.78 (0.01)	0.73 (0.02)
DNN-R4	0.90 (0.01)	0.64 (0.03)	0.50 (0.02)	0.81 (0.01)	0.76 (0.02)
XGBoost-ENN	0.95 (0.00)	0.51 (0.04)	0.48 (0.03)	0.73 (0.02)	0.69 (0.03)
XGBoost-SMOTE-ENN	0.93 (0.01)	0.55 (0.03)	0.48 (0.02)	0.74 (0.01)	0.72 (0.02)
XGBoost-KSMOTE	0.95 (0.01)	0.43 (0.03)	0.43 (0.02)	0.69 (0.01)	0.64 (0.02)

Data are presented as mean (standard deviation). MCC: Matthews correlation coefficient; ROC AUC: area under the receiver operating characteristic curve; G-Mean: geometric mean of sensitivity and specificity; DNN: deep neural network; TL: transfer learning; RNN: recurrent neural network; R4: REINVENT4; ENN: edited nearest neighbor; SMOTE-ENN: Synthetic Minority Oversampling Technique and edited nearest neighbor; KSMOTE: K-means SMOTE.

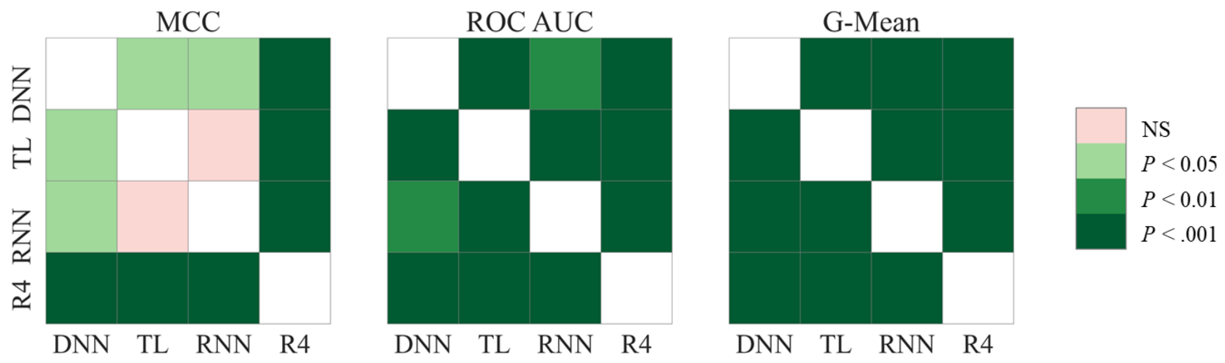

Figure S6. Statistical comparison of the DNN against the two dataset-balancing methods (TL and RNN/R4) during scaffold-split-generated test set prediction using sign plots showing the results from the Conover-Friedman test for MCC, ROC AUC, and G-Mean. MCC: Matthews correlation coefficient; ROC AUC: area under the receiver operating characteristic curve; G-Mean: geometric mean of sensitivity and specificity; DNN: deep neural network; TL: transfer learning; RNN: recurrent neural network; R4: REINVENT4; NS: not significant.

Table S7. Performance of the DNN compared with the two proposed dataset-balancing methods (TL and RNN/R4) and three traditional approaches during HTS test set prediction

Model	Sensitivity	Specificity	MCC	ROC AUC	G-Mean
DNN	0.94 (0.01)	0.08 (0.01)	0.01 (0.00)	0.52 (0.01)	0.27 (0.02)
DNN-TL	0.90 (0.02)	0.14 (0.03)	0.01 (0.00)	0.53 (0.01)	0.36 (0.03)
DNN-RNN	0.68 (0.02)	0.38 (0.03)	0.01 (0.00)	0.54 (0.01)	0.51 (0.01)
DNN-R4	0.56 (0.02)	0.56 (0.02)	0.03 (0.00)	0.57 (0.01)	0.56 (0.01)
XGBoost-ENN	0.93 (0.01)	0.09 (0.01)	0.01 (0.02)	0.51 (0.00)	0.29 (0.02)
XGBoost-SMOTE-ENN	0.90 (0.01)	0.12 (0.03)	0.01 (0.02)	0.50 (0.00)	0.32 (0.01)
XGBoost-KSMOTE	0.95 (0.01)	0.06 (0.01)	0.00 (0.00)	0.50 (0.00)	0.23 (0.02)

Data are presented as mean (standard deviation). MCC: Matthews correlation coefficient; ROC AUC: area under the receiver operating characteristic curve; G-Mean: geometric mean of sensitivity and specificity; HTS: high-throughput screening; DNN: deep neural network; TL: transfer learning; RNN: recurrent neural network; R4: REINVENT4; ENN: edited nearest neighbor; SMOTE-ENN: Synthetic Minority Oversampling Technique and edited nearest neighbor; KSMOTE: K-means SMOTE.

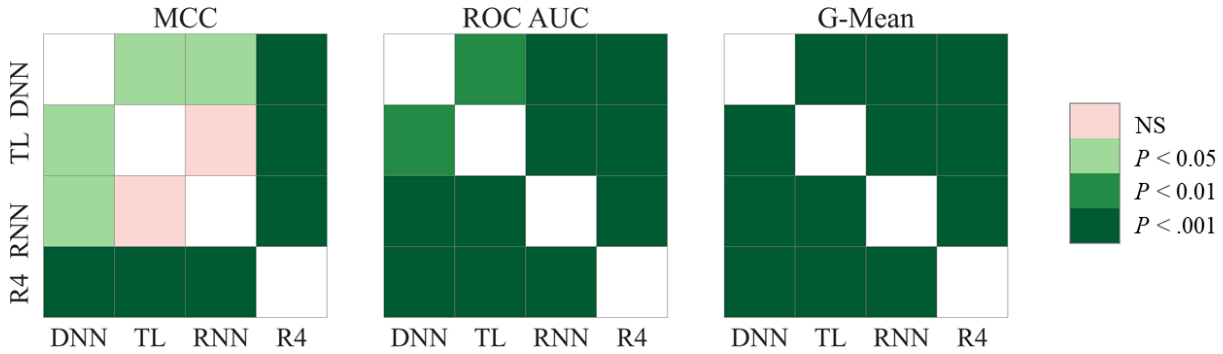

Figure S7. Statistical comparison of the DNN against the two dataset-balancing methods (TL and RNN/R4) during HTS test set prediction using sign plots showing the results from the Conover-Friedman test for MCC, ROC AUC, and G-Mean. MCC: Matthews correlation coefficient; ROC AUC: area under the receiver operating characteristic curve; G-Mean: geometric mean of sensitivity and specificity; HTS: high-throughput screening; DNN: deep neural network; TL: transfer learning; RNN: recurrent neural network; R4: REINVENT4; NS: not significant.

Table S8. Performance of the DNN compared with the two proposed dataset-balancing methods (TL and RNN/R4) during DrugBank test set prediction

Model	Accuracy
DNN	0.97 (0.00)
DNN-TL	1.00 (0.00)
DNN-RNN	0.95 (0.01)
DNN-R4	0.92 (0.02)

Data are presented as mean (standard deviation). DNN: deep neural network; TL: transfer learning; RNN: recurrent neural network; R4: REINVENT4.

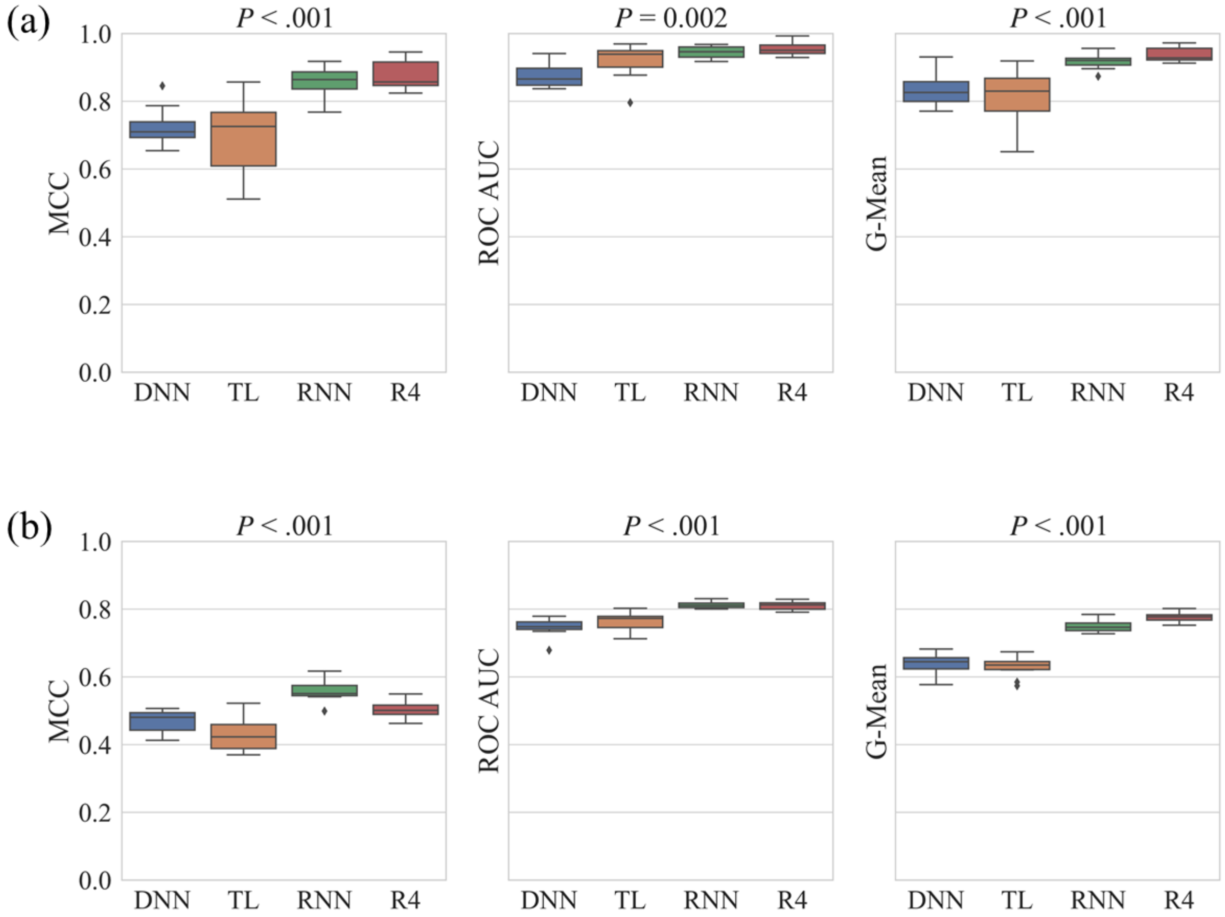

Figure S8. Boxplots comparing the performance of DNN models for beta-2 adrenergic receptor against the two dataset-balancing methods (TL and RNN/R4) during (a) 10-fold cross validation and (b) scaffold-split-generated test set for MCC, ROC AUC, and G-Mean. The *P*-value determined using Friedman's test is shown above each plot. MCC: Matthews correlation coefficient; ROC AUC: area under the receiver operating characteristic curve; G-Mean: geometric mean of sensitivity and specificity; DNN: deep neural network; TL: transfer learning; RNN: recurrent neural network; R4: REINVENT4.

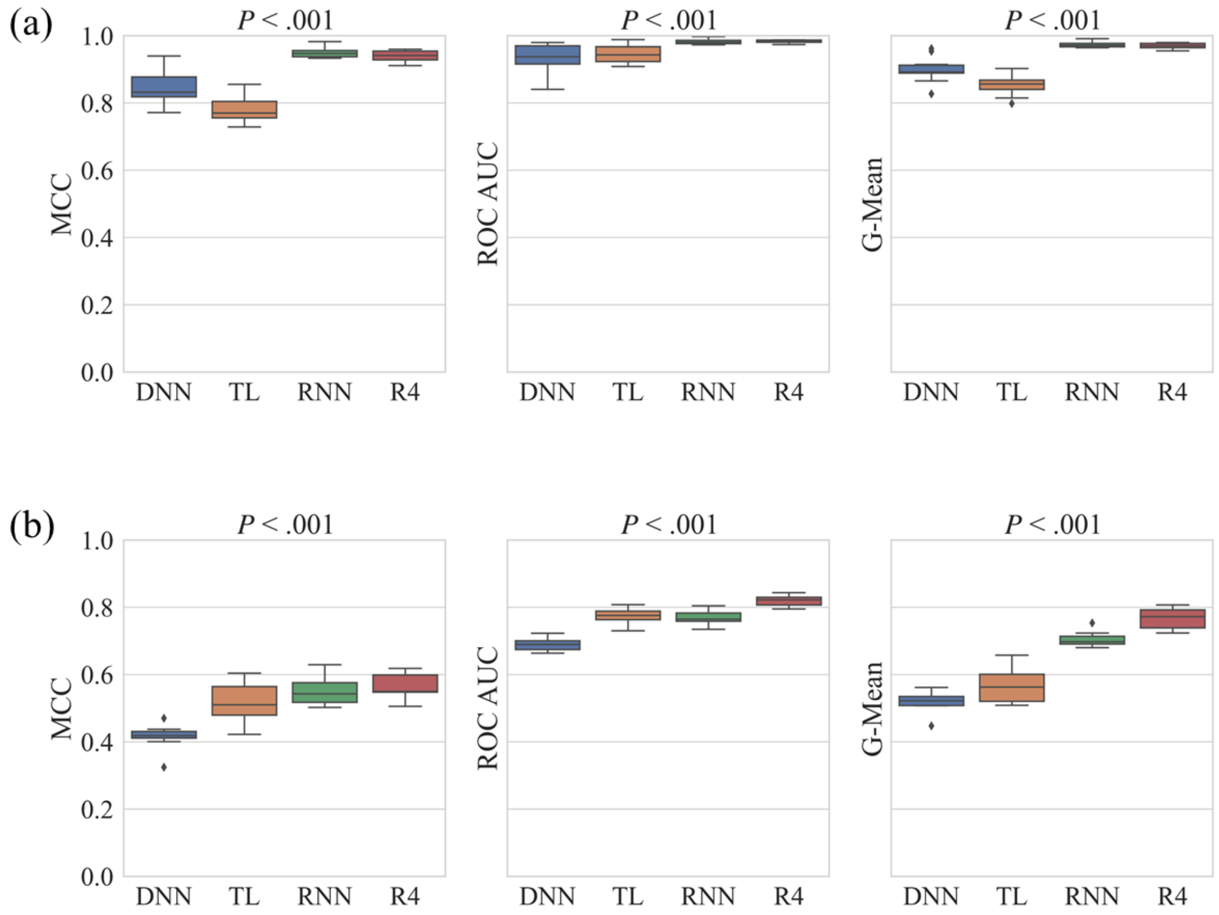

Figure S9. Boxplots comparing the performance of DNN models for adenosine receptor A2a against the two dataset-balancing methods (TL and RNN/R4) during (a) 10-fold cross validation and (b) scaffold-split-generated test set for MCC, ROC AUC, and G-Mean. The *P*-value determined using Friedman's test is shown above each plot. MCC: Matthews correlation coefficient; ROC AUC: area under the receiver operating characteristic curve; G-Mean: geometric mean of sensitivity and specificity; DNN: deep neural network; TL: transfer learning; RNN: recurrent neural network; R4: REINVENT4.

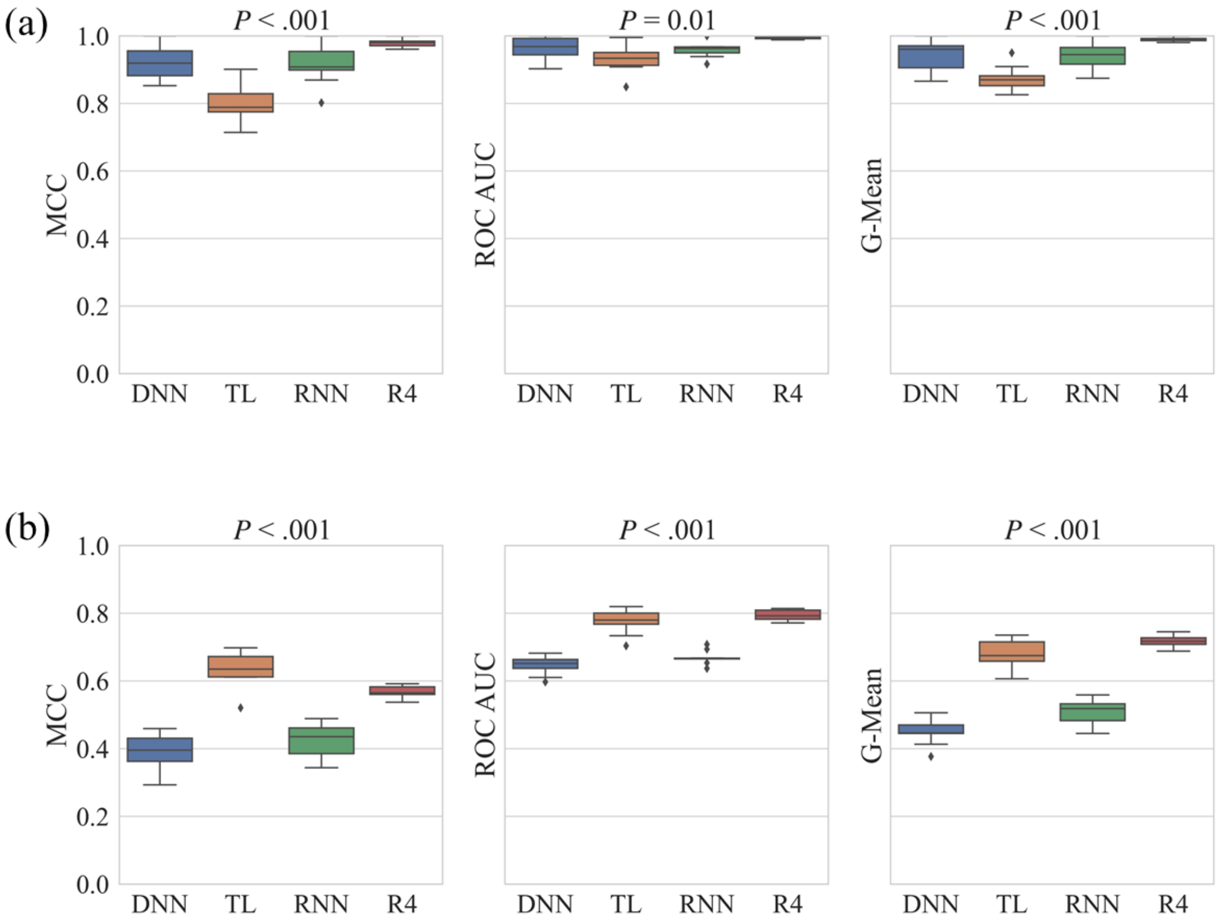

Figure S10. Boxplots comparing the performance of DNN models for C-C chemokine receptor type 5 against the two dataset-balancing methods (TL and RNN/R4) during (a) 10-fold cross validation and (b) scaffold-split-generated test set for MCC, ROC AUC, and G-Mean. The *P*-value determined using Friedman's test is shown above each plot. MCC: Matthews correlation coefficient; ROC AUC: area under the receiver operating characteristic curve; G-Mean: geometric mean of sensitivity and specificity; DNN: deep neural network; TL: transfer learning; RNN: recurrent neural network; R4: REINVENT4.

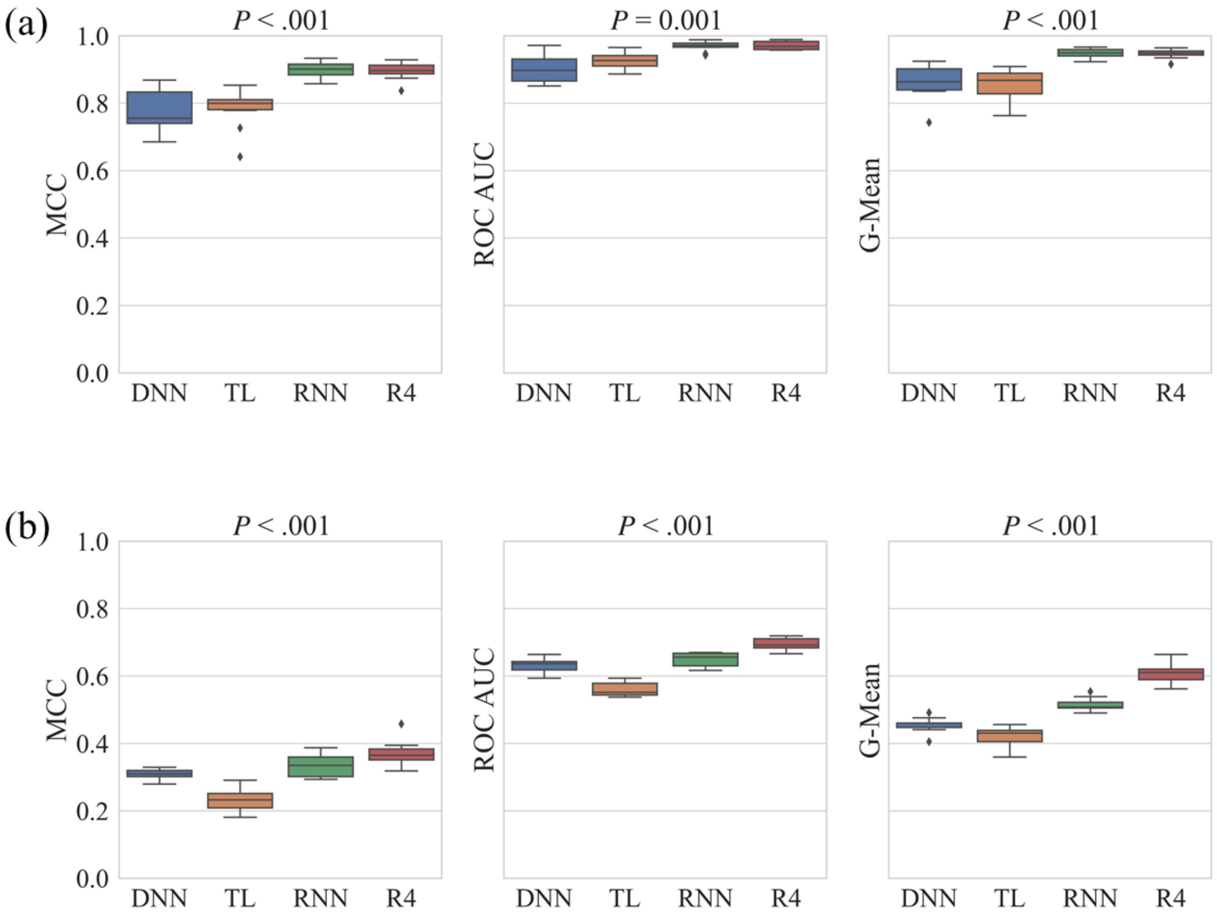

Figure S11. Boxplots comparing the performance of DNN models for metabotropic glutamate receptor 5 against the two dataset-balancing methods (TL and RNN/R4) during (a) 10-fold cross validation and (b) scaffold-split-generated test set for MCC, ROC AUC, and G-Mean. The *P*-value determined using Friedman's test is shown above each plot. MCC: Matthews correlation coefficient; ROC AUC: area under the receiver operating characteristic curve; G-Mean: geometric mean of sensitivity and specificity; DNN: deep neural network; TL: transfer learning; RNN: recurrent neural network; R4: REINVENT4.

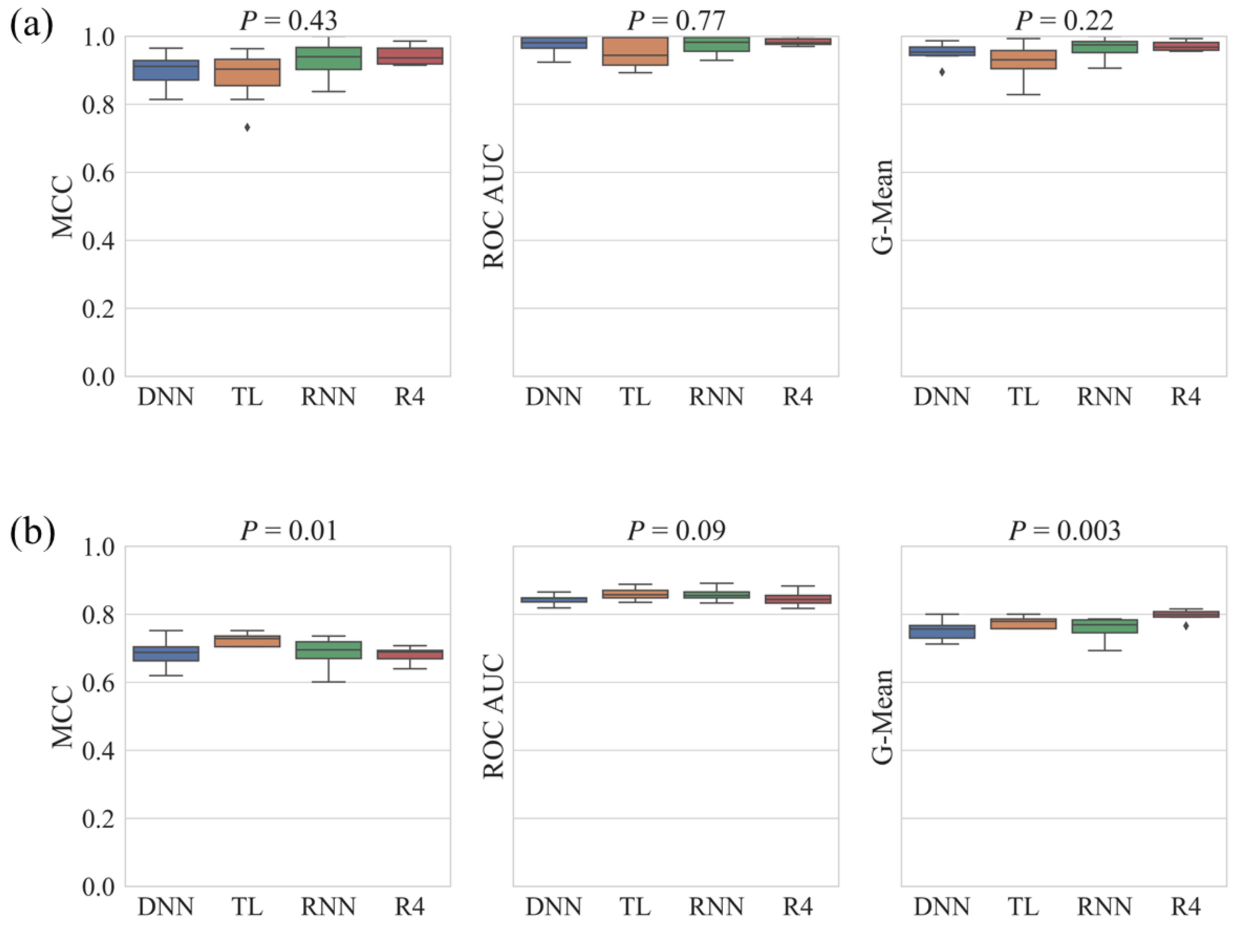

Figure S12. Boxplots comparing the performance of DNN models for gastrin/cholecystokinin type B receptor against the two dataset-balancing methods (TL and RNN/R4) during (a) 10-fold cross validation and (b) scaffold-split-generated test set for MCC, ROC AUC, and G-Mean. The *P*-value determined using Friedman's test is shown above each plot. MCC: Matthews correlation coefficient; ROC AUC: area under the receiver operating characteristic curve; G-Mean: geometric mean of sensitivity and specificity; DNN: deep neural network; TL: transfer learning; RNN: recurrent neural network; R4: REINVENT4.

References

1 Polykovskiy, D. *et al.* Molecular Sets (MOSES): a benchmarking platform for molecular generation models. *Front Pharmacol* **11**, 565644 (2020).
